# Computer-aided diagnosis of cervical dysplasia using colposcopic images

**DOI:** 10.3389/fonc.2022.905623

**Published:** 2022-08-05

**Authors:** Jing-Hang Ma, Shang-Feng You, Ji-Sen Xue, Xiao-Lin Li, Yi-Yao Chen, Yan Hu, Zhen Feng

**Affiliations:** First Affiliated Hospital of Wenzhou Medical University, Wenzhou Medical University, Wenzhou, China

**Keywords:** Cervical dysplasia, colposcopy, computer-aided diagnosis, multi-modal machine learning, feature extraction - classification ensemble

## Abstract

**Background:**

computer-aided diagnosis of medical images is becoming more significant in intelligent medicine. Colposcopy-guided biopsy with pathological diagnosis is the gold standard in diagnosing CIN and invasive cervical cancer. However, it struggles with its low sensitivity in differentiating cancer/HSIL from LSIL/normal, particularly in areas with a lack of skilled colposcopists and access to adequate medical resources.

**Methods:**

the model used the auto-segmented colposcopic images to extract color and texture features using the T-test method. It then augmented minority data using the SMOTE method to balance the skewed class distribution. Finally, it used an RBF-SVM to generate a preliminary output. The results, integrating the TCT, HPV tests, and age, were combined into a naïve Bayes classifier for cervical lesion diagnosis.

**Results:**

the multimodal machine learning model achieved physician-level performance (sensitivity: 51.2%, specificity: 86.9%, accuracy: 81.8%), and it could be interpreted by feature extraction and visualization. With the aid of the model, colposcopists improved the sensitivity from 53.7% to 70.7% with an acceptable specificity of 81.1% and accuracy of 79.6%.

**Conclusion:**

using a computer-aided diagnosis system, physicians could identify cancer/HSIL with greater sensitivity, which guided biopsy to take timely treatment.

## Introduction

With 570,000 new cases and 311,000 cases of death in 2018, cervical cancer accounts for the highest morbidity of gynecologic malignancies in women worldwide (1). However, the progress of the disease is slow, which can be prevented by detecting human papillomavirus (HPV) infection and precancerous changes (2). Cervical precancerous changes are also known as cervical intraepithelial neoplasia (CIN); according to the extent of lesion involvement, it is classified in grades: CIN1 (mild), CIN2 (moderate), and CIN3 (severe) (3). Patients with high-grade squamous intraepithelial lesion (HSIL; CINII/CINIII) are likely to progress to invasive cervical cancer and require further treatment, whereas patients with low-grade squamous intraepithelial lesion (LSIL; CINI) have a high probability of regressing (4). In clinical practice, it is crucial to differentiate cancer/HSIL from LSIL/normal to take timely treatment.

The standard screening methods for cervical cancer include ThinPrep cytologic test (TCT), human papillomavirus (HPV) tests, and colposcopy (5). TCTs are effective but require a laboratory and pathologists to evaluate the samples, and they suffer from low sensitivity in detecting cancer/HSIL (6). The HPV tests have high sensitivity in detecting cancer/HSIL but suffer from a high false-positive rate, especially in young women (6, 7). Colposcopy is a diagnostic procedure for patients with abnormal TCT or/and HPV tests. Colposcopists use a camera to take photographs of the cervix (cervicograms), with visual inspection applying 3%–5% acetic acid solution (VIA) and Lugol’s iodine (VILI) to improve visualization of the abnormal areas, which are used to guide biopsy for pathological confirmation of cervical abnormalities. Reversible coagulation in nuclear proteins and cytokeratin was caused when applying acetic acid to the cervix. Due to the high nuclear protein content in lesion areas, whitening and mosaic-textured features can be seen while normal cervix regions remain a light pink color (8). Normal cervical epithelial cells are glycogen rich, which takes up Lugol’s iodine and turns dark brown, while lesion areas are glycogen deficient, which appear pale (8). Colposcopy-guided biopsy with pathological diagnosis is the gold standard in diagnosing CIN and invasive cervical cancer; however, due to the lack of well-trained colposcopists, the poor correlation between visual and pathological diagnosis and disagreement among experts (9, 10) as well as the sensitivity and the specificity of colposcopy is not desirable enough, especially in the developing country (11–13).

Computer-aided medical diagnosis can successfully complete a variety of medical tasks by efficiently exploring the essence of a large amount of clinical data. The colposcopy-guided cervical biopsy is essential for detecting CIN in cervical cancer screening, but there are difficulties with increasing sensitivity globally. Pilot studies used the k-nearest neighbor (K-NN) algorithm (14) and the opacity index (15) to observe the aceto-white patterns in the VIA screening to distinguish between normal and abnormal cervices. Statistical analysis was used to characterize the degree of cervical lesions with color (16, 17) or texture features (18–21). The extracted features from time-lapsed VIA images were combined using a graph convolutional network with edge features (22). Deep learning networks were also used to complete the tasks, but the predictive power was limited by the small training sets (about 100 patients) (23, 24). Another deep network-based literature had a sizable dataset, but its labels were based on the physician’s subjective diagnosis rather than the ground truth (25). In addition, the black box of the models hardly helps with cervical biopsy guidance.

These aforementioned algorithms shared a common drawback. They were not resistant to noises using just VIA images. The result was lack of diagnostic confidence due to the imaging quality and normal epithelium shades. Compared to VIA alone, co-testing with VILI appeared to boost performance (26). A neural network architecture for the combination of VIA and VILI images was suggested (27), and a feature extraction-based machine learning algorithm was developed (28). TCT, HPV tests, and some clinical data, in addition to information from the VIA and VILI images, help identify cervical lesions from various angles. They have the potential to be fully utilized by a diagnosis system to identify cervical lesions.

In this work, we collected VIA and VILI images and clinical information from each of the 1361 patients, and the gold standard of pathologic diagnosis was used as the ground truth. The model used an SVM with radial basis function kernel (RBF-SVM) to generate a preliminary output after extracting color and texture features from the cervical regions that were automatically segmented. The output of the first stage was passed on to the second stage input, which combined the TCT, HPV test, and age, to build a naïve Bayes algorithm for cervical lesion diagnosis. This model’s performance was compared to that of colposcopists and other machine learning models. The visualized interpretable features help with biopsy by identifying potential lesion sites.

## Methods

### Dataset

We gathered clinical data, TCTs, HPV tests, and cervical images of colposcopy (TR6000C) from 1,361 patients (ages ranged from 16 to 83) at the First Affiliated Hospital of Wenzhou Medical University in China during the period from 1 August to 31 November 2020 for this study. Each patient signed a consent authorizing colposcopy with biopsy. The colposcopy examination was carried out by nine physicians with specialized knowledge, including one chef physician, four attending physicians, and four resident physicians. Physicians made a diagnosis for each patient based on the cervigrams. The pathological result of cervical biopsy served as the ground truth to identify the degree of cervical lesions.

Among 1,361 patients, we eliminated 229 patients with blurred images or unclear exposed cervix portion (such as cervix obscured by instrument, contraception tail wire, and blood or cervix obscured by vaginal sidewalls and speculums greater than 25%), 71 patients with a history of hysterectomy or cervical operation (such as cryotherapy, laser therapy, loop electrosurgical excision procedure (LEEP), or cold knife conization (CKC)), and 75 patients with information loss (i.e., no TCT or HPV tests, no biopsy). After filtering the images, the study included a total of 986 patients and 1,972 cervical images (each patient had one VIA image and one VILI image). These patients were categorized into normal (288,29.2%), LSIL (561,56.9%), HSIL (124,12.6%), and cancer (13,1.3%) according to pathological results. The training set consisted of 701 patients from the first 3 months, and the test set consisted of the additional 285 patients from the fourth month.

HPV tests used in this study were HPV DNA Test (Tellgenplex HPV 27 Genotyping Assay). The results of HPV tests were divided into positive (885, 89.8%) and negative (101, 10.2%). HPV positive was subdivided into three classes (1): HPV 16/18 positive (2), high-risk (non-16/18) HPV positive, and (3) low-risk HPV positive. Multiple positive options were permitted for patients who had multiple HPV infections. The Bethesda 2014 classification was used to divide the TCTs into six categories (29). **Table 1** displays the distribution of TCT and HPV tests.

**Table 1:** The distribution of HPV tests and TCT tests in the 986 patients.

**HPV**	N%	**TCT**	N%
HPV 16/18 positive	27.7%	NILM	51.6%
High-risk (non-16/18)	65.8%	ASCUS	24.1%
HPV positive		ASC-H	3.8%
Low-risk HPV positive	10.4%	LSIL	15.2%
HPV negative	10.2%	HSIL	4.4%
		AGC	0.9%

### Colposcopic image segmentation

We defined the region of interest with a minimum rectangle in the vaginal wall around the cervix and conducted a further investigation because vaginal sidewalls and speculums affected the identification of cervical lesions. We used the transfer learning technique to pretrain the weights of five different deep learning architectures (DenseNet-169, ResNet-50, ResNet-101, VGG-16, and Xception). Four neurons representing width, height, and the coordinates of the left-bottom endpoint were used in place of the classification head. Physicians annotated 200 original VIA and 200 original VILI images with bounding boxes around the cervix to serve as the ground-truth labels, and they were divided into training, validation, and test sets with a 120/30/50 split, respectively.

**Figure 1:**
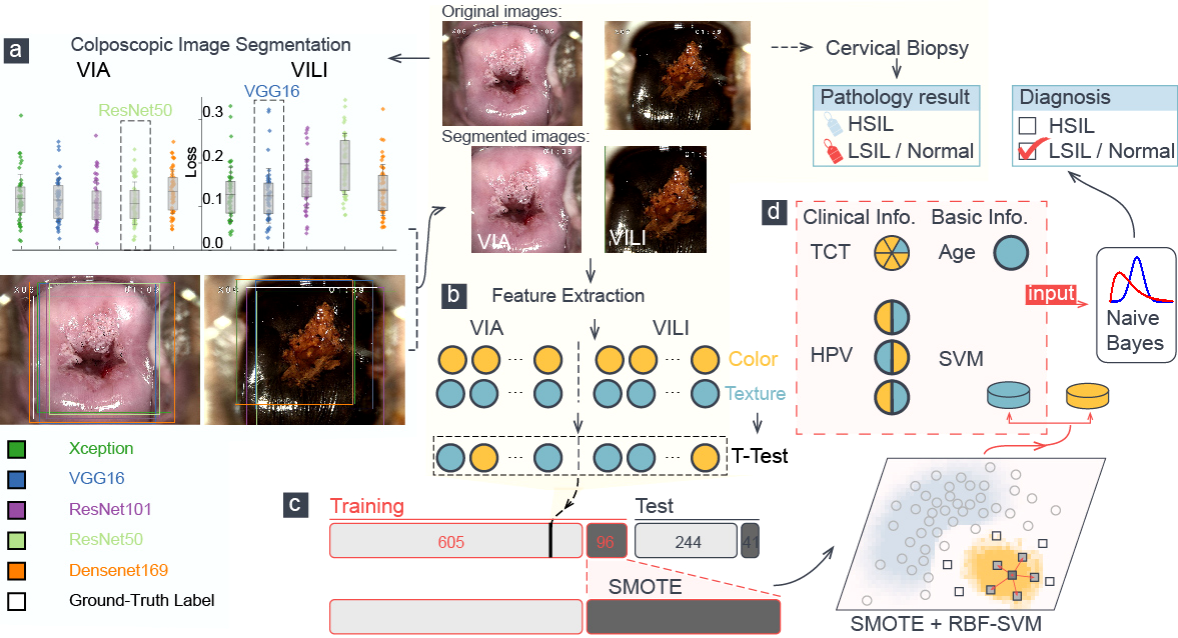
A schematic representation of the training procedure of our model. **(A)** Loss analysis of five segmentation algorithms was presented, and one sample was visualized. Each color attribute represented a different algorithm, and the white rectangular outline showed the ground truth of segmentation. **(B)** Color and texture features in segmented VIA/VILI images were selected and were further extracted using t-test. **(C)** The H-group was augmented using the SMOTE algorithm and were then fed into an RBF-SVM for training. **(D)** Six features went into the naïve Bayes classifier to perform the final classification, which was compared with the pathological result.

In order to assess the performance of the five deep learning models, we calculated the mismatch loss of the 50 images between the predicted regions and the ground truth in the testing stage. According to the mean and standard deviation of loss, the four models highlighted in bold in **Figure 1A** and **Table 2** show comparably good results. Further reviewing the performance from a medical standpoint, physicians found that ResNet-50 performed best for VIA image segmentation because it more accurately detected the cervix. Similar to that, VGG-16 worked well for segmenting 200 labeled VILI images, with the results displayed in **Table 2**.

**Table 2:** Loss analysis of five deep learning algorithms for image segmentation.

	VIA	VILI
DenseNet-169	0.13 ± 0.05	**0.14** ± **0.06**
ResNet-50	**0.10** ± **0.05**	0.20 ± 0.07
ResNet-101	**0.11** ± **0.05**	0.15 ± 0.05
VGG-16	**0.12** ± **0.05**	**0.13** ± **0.06**
Xception	**0.12** ± **0.05**	**0.13** ± **0.06**

Bold-type values indicated algorithms with relatively low loss.

The underlined had an optimal clinical performance.

Sample size: 120/30/50 (training/validation/test set)

All VIA and VILI images were segmented using the two well-trained deep learning models. Physicians then double-checked them and made any necessary corrections to those with a significant deviation (more than about 10%). In fact, only 4.45% VIA and 2.50% VILI images were altered by physicians, confirming the models’ validity. Pixels were used to measure the width and height of each image. The cropped images have an average size of (905 ± 66) × (866 ± 101) for the original images (sized 144 × 1,080).

### Color features

The lesion localization is determined mainly according to the VIA and VILI images. A crucial diagnostic tool for cervical dysplasia is color features. In order to fully characterize the color features of an image, a color space is used to represent color that can be reproduced on an image. The most popular color space is RGB, but it completely ignores the environment’s lighting and the camera’s sensitivity, taking the pixels in a digital image at face value. With its foundation in hue, saturation, and lightness, the hue–saturation–value (HSV) space is more perceptually relevant and intuitive. A grayscale is a color space that displays an RGB image’s luminance data. A fairly logical way to arrange colors is from black to white (luminance: Ll),green to red (La), and blue to yellow (Lb) (CIELAB). In this space, colors that are separated by the same amount appear to have approximately equal differences. Luminance, Chroma blue, and Chroma red (YCbCr) occupy a different space, where Luminance (the Y component) denotes the color’s brightness and Cb and Cr denote the blue and red components, respectively, in relation to the green component (30).

We calculated the statistical dispersion with the standard deviation and identified central tendency with the mean, median, and mode in order to investigate the statistical characteristics of each color channel. To automatically determine a threshold value and reduce the weighted within-class variance, we also used the Otsu thresholding algorithm (31). To describe each VIA and VILI image, a total of 65 color features were extracted.

### Haralick texture features

An image’s texture is defined as the regular distribution of patterns and colors. When acetic acid is applied to a cervix with lesions, different abnormal epithelium textures, such as textured mosaicism, punctation, and vascular structures, are frequently visible (20). Haralick texture, a second-order statistical technique for calculating the spatial relationship, searches for pairs of adjacent pixel values in an image using the gray-level co-occurrence matrix (GLCM) and records them across the entire image (32, 33).

The pixel pair distance offsets of 1, 5, 10, and 15 pixels were used to compute the GLCMs before arriving at the four features within each offset. The GLCM’s default adjacency calculation was done from left to right (0 in degree), but the texture features actually tended to be dispersed throughout all directions. Additionally, we took note of the other three pixel-pair directions (diagonal, vertical, and anti-diagonal, or 45°, 90°, and 135° in degree) and averaged them to determine the features. Six Haralick features—angular second moment (ASM), contrast, correlation, dissimilarity, energy, and homogeneity—were extracted from these GLCMs in each pixel offset, resulting in 24 texture features for each image. The characteristics were formulated mathematically:

Contrast: $\sum_{i,j=0}^{levels-1}P_{i,j}{\left(i-j\right)}^{2}$

Dissimilarity: $\sum_{i,j=0}^{levels-1}P_{i,j}\left|i-j\right|$

Homogeneity: $\sum_{i,j=0}^{levels-1}\frac{P_{i,j}}{1+{\left(i-j\right)}^{2}}$

*P* is the GLCM value, and *P*[*i*, *j*, *d*, *θ*] indicates the number of times that gray-level *j* occurs at a distance of d an angle of *θ* from gray-level *i*.

### Performance metrics

Sensitivity measures the percentage of cancer/HSIL (H-group) patients who are correctly identified, and specificity is the extent to which LSIL/normal (LN-group) ones are correctly identified as such. Accuracy calculates the correct prediction percentage all over samples. In binary, unbalanced classification tasks, the area under the precision-recall curve (AUPRC) is frequently used. The macro-averaged F1 score, which can be calculated as follows, is the arithmetic mean of the per-class F1 scores and balances sensitivity and specificity:


$$F1_{Macro}=\frac{1}{N}\sum_{i=1}^{N}\frac{2\times Sen_{i}\times Spec_{i}}{Sen_{i}+Spec_{i}}$$

where *Sen_i_* and *Spec_i_* are the sensitivity and specificity for the *i^th^* class.

The macro-averaged F1 score equally weighs each class’s sensitivity and specificity. It is an appropriate metric for thoroughly assessing model performance. Sensitivity is set as one of the key performance metrics because it is of the utmost clinical importance to identify patients with severe cervical lesions.

### Feature selection

A single VIA or VILI image yielded 24 Haralick texture features and 65 color features. Due to redundancy and a greater emphasis on noise in machine learning algorithms, an excessive number of features resulted in a failed classification, though. To identify features that significantly differed between the means of the two classes, a T-test was used. We used the T-test to analyze the features in 701 VIA training images, and then we sorted them according to their adjusted P-values. Similar to that, VILI image features were also sorted. We used various combinations of the top-ranked features to feed the model, and we used the entire training process described in subsection 2.7. To assess all performance metrics and choose the best VIA and VILI feature combination, the macro-averaged F1 score was used.

### Multimodal machine learning for identifying cancer/HSIL patients

It was common that H cases (cancer/HSIL) were much fewer than LN ones (LSIL/normal), and in this work, H cases accounted for 137 out of 986. The machine learning algorithm would have a tendency to predict the majority in a skewed class distribution. Undersampling reduces the majority samples and achieves a balanced class proportion, wasting valuable medical data in the process. By duplicating minority data, the oversampling technique increases the sample size of the minority class (34). Despite being balanced, the class distribution adds no fresh data or variation to the model. A K-NN algorithm is used in the oversampling technique variant known as Synthetic Minority Oversampling Technique (SMOTE) to generate artificial data. The parameter k was set to 2 in this work to create synthetic samples (35).

Using a support vector machine (SVM) classifier, which maps data onto points in a high-dimensional space and finds an ideal hyperplane to divide data into classes, we increased the H cases to achieve a similar proportion of LN ones. Using the balanced augmented data, we trained an SVM with the radial basis function (RBF) kernel. The kernel was expressed as follows: exp(−*γ*∥ *x*−*x* ^′^ ∥^2^) where *γ* was 0.72. The other parameter, C, was set to 0.12 as a compromise between the training samples’ misclassification and the simplicity of the decision surface.

The class probability estimates were produced by the RBF-SVM as the initial classification results. Together with the HPV, TCT, and patient age, a second diagnosis was made. Therefore, using the six input features listed in **Table 3**, we built a naïve Bayes classifier to perform the final classification.

**Table 3:** Summary of features for the naïve Bayes classifiers.

	Feature	Type	Range
1	SVM output	Numerical	[0, 1]
2	Age	Numerical	[16,83]
3	HPV-1^1^	Categorical	0,1
4	HPV-2^2^	Categorical	0,1
5	HPV-3^3^	Categorical	0,1
6	TCT	Categorical	1,2,3,4,5,6 ^4^

^1^:HPV 16/18 positive; ^2^: high-risk (non-16/18) HPV positive; ^3^: low-risk HPV positive.

^4^ : 1: NILM; 2: ASCUS; 3: ASC-H; 4: LSIL; 5:HSIL; 6:AGC.

## Results

### Feature extraction and feature selection

**Figure 1** depicts a schematic diagram of our model’s training process. The ResNet-50 and VGG-16 deep learning algorithms automatically segmented the original VIA and VILI images. Sixty-five color features and 24 Haralick texture features were extracted from the cropped VIA or VILI image, and these features were then ranked in ascending order based on their p-values from T-test analysis. **Table 4** contains the top 10 features from VIA and VILI images, where VIA features were made up of four color features and six texture features while VILI features were all color-related. This result is consistent with the findings that Lugol’s iodine only contributes to color contrast while acetic acid causes both texture modification and color contrast (8).

**Table 4:** Top-ranked VIA/VILI features between H– and LN– groups.

VIA feature	H (N = 96)	LN (N = 594)	P-value
Dissimilarity (15)^ζ^	12.80 ± 3.54	11.36 ± 2.67	2.33 ×10^-4^
Dissimilarity (10) ^ζ^	10.59 ± 2.94	9.46 ± 2.29	5.07 × 10^-4^
Std (Lb) ^ζ^	6.85 ± 2.31	5.99 ± 1.63	7.44 × 10^-4^
Std (S) ^ζ^	26.01 ± 10.83	22.12 ± 6.17	8.61 × 10^-4^
Std (Cb) ^ζ^	6.31 ± 2.14	5.55 ± 1.48	1.01 × 10^-3^
Contrast (15)	486.72 ± 265.95	392.07 ± 181.19	1.12 × 10^-3^
Std (La)	4.93 ± 1.77	4.33 ± 1.22	2.09 × 10^-3^
Homogeneity (15)	0.11 ± 0.02	0.12 ± 0.02	2.31 × 10^-3^
Contrast (10)	373.02 ± 198.80	307.16 ± 148.59	2.50 × 10^-3^
Dissimilarity (5)	8.01 ± 2.22	7.28 ± 1.81	2.92 × 10^-3^
VILI feature	H (N=96)	LN (N=594)	P-value
Std (Lb) ^ζ^	12.72 ± 3.53	9.89 ± 3.20	2.45 × 10^-11^
Std (Cb) ^ζ^	12.38 ± 4.13	9.21 ± 3.40	9.97 × 10^-11^
Otsu (Lb) ^ζ^	103.39 ± 8.10	109.13 ± 7.41	1.96 × 10^-9^
Std (La) ^ζ^	6.61 ± 2.59	4.87 ± 2.06	7.22 × 10^-9^
Std (Cr) ^ζ^	8.51 ± 2.79	6.64 ± 2.18	7.59 × 10^-9^
Otsu (Cb)	151.56 ± 8.73	145.74 ± 7.63	1.10 × 10^-8^
Otsu (La)	135.73 ± 5.06	132.34 ± 4.61	1.13 × 10^-8^
Mean (Lb)	105.88 ± 11.33	12.39 ± 8.76	4.42 × 10^-7^
Mean (Cb)	149.10 ± 11.29	142.67 ± 8.30	4.42 × 10-7
Otsu (Cr)	112.64 ± 6.12	115.84 ± 5.22	4.10 × 10^-6^

ζ: Features are used in our model, that is, five top-ranked features.

The pixel pair distance offsets or the color channel is inside the parentheses.

Sd, standard deviation; Otsu, Otsu thresholding.

**Figure 2:**
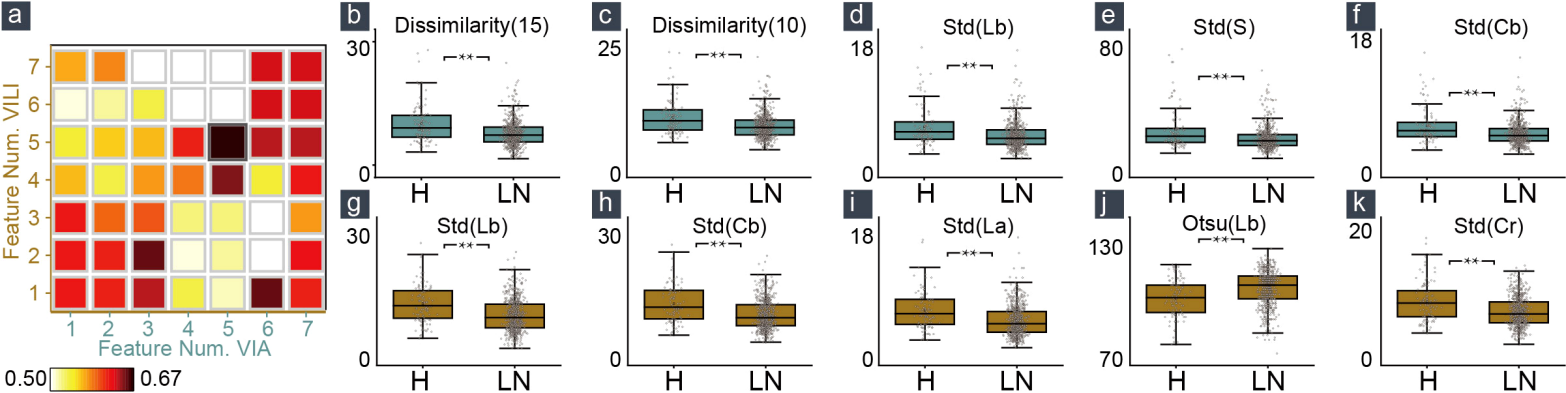
Feature selection and feature distribution visualization. **(A)** The combination of five VIA and five VILI features achieved the maximum macro-averaged F1 score. Statistical distribution of the selected features in VIA **(B–F)** and VILI **(G–K)** training samples.

We calculated the average of the macro-averaged F1 score over more than five entries to determine the ideal number of features extracted from VIA and VILI images using various combinations of the top-ranked features and clinical data as input to our multimodal machine learning model. The maximum macro-averaged F1-score of 0.67 was attained by the combination of five VIA features and five VILI features, as shown in **Figure 2A**.

**Figure 3:**
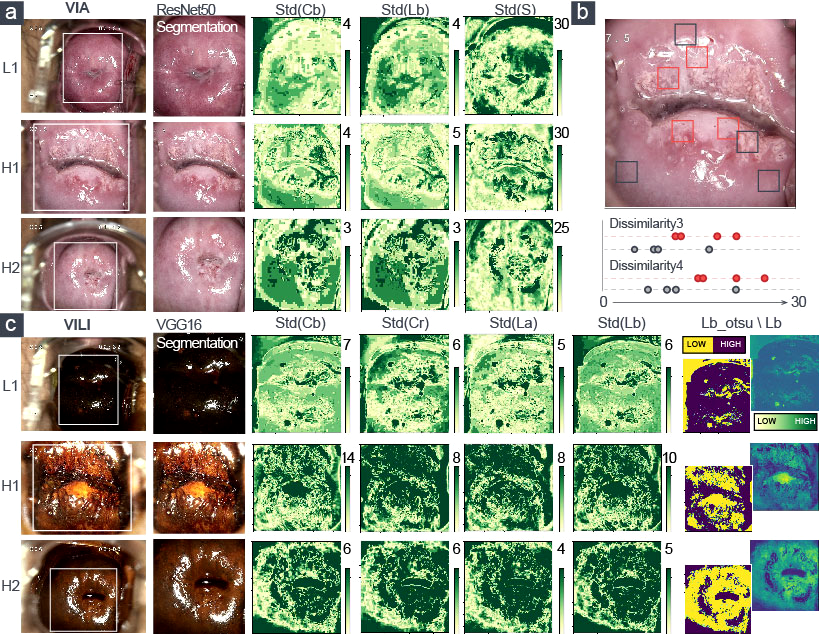
Visualization of the extracted five top-ranked features of VIA and VILI images. LN-group: L1; H-group: H1, H2. **(A)** Three color features and **(B)** two texture features were for VIA images. **(C)** Five color features were for VILI images.

In **Figures 2B–K**, we also showed how the training data’s feature distribution was distributed. For VIA, the dissimilarity feature had a noticeable difference with 15- and 10-pixel offsets. It remained high in the H–group, indicating a high contrast in the area with the most severe lesions. The three color channels’ standard deviations (Lb, S, and Cb) were significantly higher in the H–group, which resulted in more fluctuations in the group’s blue-difference chroma signals and saturation components. For VILI, the four color channels (Lb, Cb, La, Cr) had significantly higher standard deviations in the H–group, which indicated more fluctuations in the blue- and red-difference color components. Additionally, the Otsu threshold of CIELAB-Lb showed that the H–groups were more likely to contain more yellow components and fewer blue ones. We used one LN case and two H cases as examples to visualize the VIA (**Figures 3A, B**) and VILI features for better interpretation (**Figure 3C**).

### Experimental results

The test set included all 285 patients with pathologic diagnoses as the ground truth that had never been trained. The multimodal model generated the preliminary output by applying feature extraction and RBF-SVM optimized by the SMOTE algorithm to the VIA and VILI images. The multimodal model then concatenated the HPV, TCT tests, and age to the naïve Bayes classifier for cervical-lesion diagnosis. A case entered our model’s final output and was categorized into the H–group or the LN–group, as shown in **Figure 1**.

Our model achieved a sensitivity of 51.2% with accuracy 81.8%, specificity 86.9%, and AUPRC 0.882 ± 0.014. To assess the performance, we ran a number of in-depth experiments. Features were used as the input when creating machine learning classifiers like random forest, neural network (NN), and 1D-convolutional neural network (CNN). RBF-SVM algorithms were also performed with three input configurations—VIA images only, VILI images only, and VIA and VILI images. Additionally, the SMOTE algorithm could be integrated in RBF-SVMs to balance the skewed class distribution.

All of the machine learning models mentioned above were optimized. The Gini impurity was used by the random forest classifier as the splitting criterion, and 100 decision trees were used to generate the out-of-bag predictions. Neural networks used the Adam optimization algorithm and the three-layer 10–25–2 configuration with tanh activations. The predictive threshold was indicated by the number in parenthesis after NN. A 1D-convolution layer with 64 1 × 8-sized feature maps, a max-pooling layer, and two fully connected layers made up the 1D-CNN.

Modern CNN architectures like ResNet-50 and VGG-16 were frequently utilized to carry out image classification tasks. We used transfer learning to train the classification head of the ResNet-50/VGG-16 models for VIA/VILI images to identify H-group patients because these two models showed excellent performance in identifying the cervix. For further diagnosis, we also fed the naïve Bayes classifier the output and clinical data. We implemented five models with the best parameter configurations for each of the 15 algorithms listed above, and we averaged the results in **Table 5**.

**Table 5:** Experimental results of different machine learning algorithms and performance of physician diagnoses.

	VIA	VILI	Clin.	Smo.	Sensitivity	Accuracy	Specificity
**Ours**	*	*	*	*	**51.2%**	81.8%	86.9%
Random forest	*	*			5.4%	84.0%	97.0%
NN(0.5)	*	*			7.3%	85.3%	97.5%
NN(0.8)	*	*			12.2%	82.1%	94.7%
1D-CNN	*	*			2.4%	85.0%	99.0%
RBF-SVM	*				3.9%	85.0%	98.9%
RBF-SVM		*			4.9%	81.0%	94.2%
RBF-SVM	*	*			13.2%	85.0%	97.0%
RBF-SVM	*			*	20.0%	75.0%	84.9%
RBF-SVM		*		*	8.3%	78.0%	89.3%
RBF-SVM	*	*		*	25.9%	81.0%	80.8%
ResNet-50	*				7.30%	80%	92.20%
ResNet-50+NB	*		*		17.10%	81.10%	91.80%
VGG-16		*			7.32%	86.30%	99.60%
VGG-16+NB		*	*		24.40%	86.00%	96.30%
ResNet-50+VGG-16+NB	*	*	*		29.30%	80%	88.50%
Chef physicians (88)	*	*	*		60.0%	85.2%	88.5%
Attending physicians (558)	*	*	*		59.0%	85.8%	90.2%
Resident physicians (340)	*	*	*		55.1%	86.2%	91.4%
Physicians-test set (285)	*	*	*		53.7%	84.6%	89.8%
**Ours+Physicians**	*	*	*	*	70.7%	79.6%	81.1%

*: The model or physicians used this kind of training data. Clin, Clinical information; Smo, Smote.

Eighty-eight (8.9%), 558 (56.6%), and 340 (34.5%) out of the 986 patients had diagnoses from the chef physicians, attending physicians, and resident physicians, respectively. Their performance and the number of diagnosed cases are listed in **Table 5**. Besides, in an effort to create comparable experimental conditions, we evaluated the physicians’ diagnostic performance for the same test set. In general, physicians’ sensitivity for cancer/HSIL detection ranged from 53.7% to 60.0%. Our model effectively and efficiently combined VIA and VILI images and clinical data to produce predictions with the power comparable to that of physicians.

**Figure 4:**
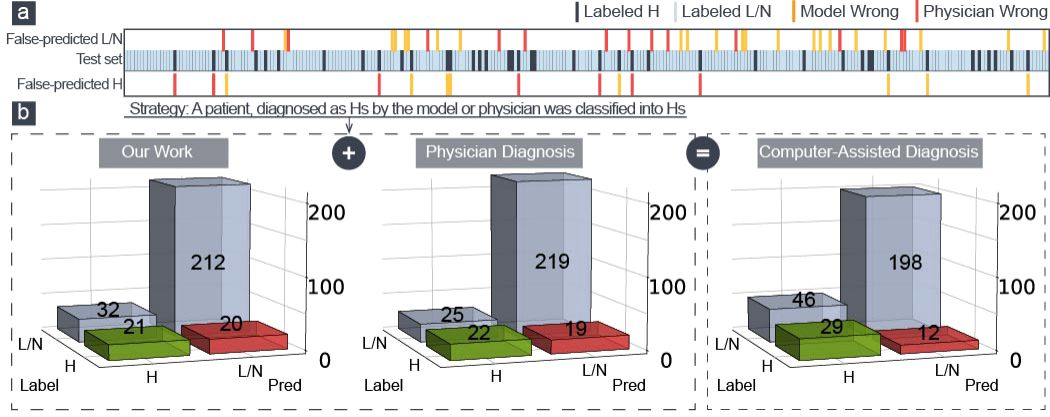
**(A)** Two hundred eighty-five patients in the test set were made wrong predictions only by physicians (red) or model (yellow). **(B)** The confusion matrix of our model, the physician diagnosis, and the model-aided physician diagnosis.

According to **Figure 4A**, this model and physicians distinguished between various patients in the H–group, demonstrating that the algorithm could identify the characteristics of severe cervical lesions that were challenging to find with the naked eyes. We introduced a computer-aided diagnosis system in colposcopy to further improve the identification of H–group patients: a patient was classified into the H–group if the model or physician diagnosed them with cancer/HSIL. The sensitivity was increased from 53.7% to 70.7% using this simple strategy, as shown in **Figure 4B**, with a respectable specificity of 81.1% and an accuracy of 79.6%. This could aid colposcopy diagnosis and biopsy in clinical practice.

## Discussion

In this study, we presented an algorithm for diagnosing cervical dysplasia using VIA and VILI images and clinical data of age, TCT, and HPV tests. The multimodal machine learning algorithm extracted the color and texture features to implement a SMOTE-based RBF-SVM model. Combining the clinical information, the algorithm achieved a sensitivity, specificity, and accuracy of 51.2%, 86.9%, and 81.8%, respectively, and physicians’ sensitivity, specificity, and accuracy were on average 53.7%, 89.8%, and 84.6%, respectively. The performance of the algorithm was comparable to physicians. In recent high-quality studies, the sensitivity and specificity to detect HSIL varied greatly, ranging between 33%–93% and 53%–95%, respectively (36–40), which was comparable to the result of our study.

We combined the cervical images of VIA and VILI to enhance the ability to recognize cervical lesions in comparison to earlier studies on cervical image analysis (19, 20, 22, 23, 27, 28). To increase sensitivity and accuracy, we added TCT, HPV tests, and age, which brought the diagnosis process closer to that of a physician. We took pathology as the ground truth, which had greater clinical significance. Additionally, the sensitivity of cancer/HSIL detection increased with the joint diagnosis of physicians and the algorithm, which was higher than with physicians or the algorithm alone. It makes sense in clinical practice to avoid unnecessary invasive operation (biopsy and diagnostic surgery) and contributes to the popularization of colposcopy in local hospitals without sufficient professionals.

The skewed class distribution made training and testing difficult, which always made the machine learning algorithms like the aforementioned random forest, neural network, and SVM to predict the majority of outcomes. Traditional oversampling techniques only replicated the minority data, which prevented the machine learning model from receiving any new data or variation. The K-NN algorithm was used by the SMOTE strategy to generate artificial data from the minority and balance the classes. Thus, as shown in **Table 5**, the sensitivity was enhanced. TCT, HPV tests, and the ages of the patients were significant reference indices in colposcopy. They were properly integrated with VIA and VILI image features by the naïve Bayes algorithm, which produced the physician-level diagnosis. A naïve Bayes classifier outperformed more complex models with smaller datasets because medical images are scarce and expensive. Besides, the model learned to recognize potential lesion sites through the 10 visualized features in our model that were extracted from VIA and VILI images, which helped guide biopsy.

This study has several limitations, including the following: first, because the cervical canal is not visible in cervical images, the algorithm is unable to accurately identify lesions in patients with lesions in the canal; second, the imbalance of data distribution (H:LN ≈ 1:7) affected the sensitivity to some extent; third, the estimated probability in the naïve Bayes classifier is inaccurate to some extent because of the naïve assumption; and fourth, the system is evaluated on the data from one single hospital, since the colposcope’s illumination characterization and imaging specifications vary with each individual colposcope equipment or environment.

## Data availability statement

The raw data supporting the conclusions of this article will be made available by the authors, without undue reservation.

## Ethics statement

The research protocol and colposcopic images, clinical information of patients used in this study were reviewed and approved by the Ethics Committee of First Affiliated Hospital of Wenzhou Medical University, Wenzhou, China[2021-zz-155]. The Ethics Committee also granted a waiver for informed consent of images and clinical information,as no biological sample was involved, no privacy of patients was included and it was a retrospective observational study.

## Author contributions

All authors contributed to the concept, design, and drafting of the study. J-HM designed the survey and wrote the manuscript. S-FY and ZF designed the model and analyzed the data. Y-YC collected the data. J-SX and X-LL critically reviewed the data and annotated images. YH Guided the research. YH and ZF critically reviewed the data, manuscript, and gave final approval. All authors contributed to the article and approved the submitted version.

## Acknowledgments

We thank Dr. Huan Hu in XMU for helpful discussions. We also thank the cervical diagnosis and treatment group, Department of Gynecology, The First Affiliated Hospital, for their statistical assistance, including Dr. Z-Z Shi, Dr. B-Y Huang, Dr. QZ, Dr. Y-L Wang, Dr L-L Chen, Dr R-Y Zheng, and Dr. Q-Y Zhu.

## Conflict of interest

The authors declare that the research was conducted in the absence of any commercial or financial relationships that could be construed as a potential conflict of interest.

## Publisher’s note

All claims expressed in this article are solely those of the authors and do not necessarily represent those of their affiliated organizations, or those of the publisher, the editors and the reviewers. Any product that may be evaluated in this article, or claim that may be made by its manufacturer, is not guaranteed or endorsed by the publisher.
